# Environmental Levels of the Antiviral Oseltamivir Induce Development of Resistance Mutation H274Y in Influenza A/H1N1 Virus in Mallards

**DOI:** 10.1371/journal.pone.0024742

**Published:** 2011-09-12

**Authors:** Josef D. Järhult, Shaman Muradrasoli, John Wahlgren, Hanna Söderström, Goran Orozovic, Gunnar Gunnarsson, Caroline Bröjer, Neus Latorre-Margalef, Jerker Fick, Roman Grabic, Johan Lennerstrand, Jonas Waldenström, Åke Lundkvist, Björn Olsen

**Affiliations:** 1 Section of Infectious Diseases, Department of Medical Sciences, Uppsala University, Uppsala, Sweden; 2 Section of Bacteriology and Food Safety, Department of Biomedical Sciences and Veterinary Public Health, Swedish University of Agricultural Sciences, Uppsala, Sweden; 3 Swedish Institute for Infectious Disease Control and Karolinska Institute, Microbiology and Tumor Biology Center, Stockholm, Sweden; 4 Department of Chemistry, Umeå University, Umeå, Sweden; 5 Section for Zoonotic Ecology and Epidemiology, School of Natural Sciences, Linnaeus University, Kalmar, Sweden; 6 Aquatic Biology and Chemistry Group, Kristianstad University, Kristianstad, Sweden; 7 National Veterinary Institute, Uppsala, Sweden; 8 Section of Pathology, Pharmacology and Toxicology, Department of Biomedical Sciences and Veterinary Public Health, Swedish University of Agricultural Sciences, Uppsala, Sweden; 9 Research Institute of Fish Culture and Hydrobiology, Faculty of Fisheries and Protection of Waters, University of South Bohemia in Ceske Budejovice, Vodnany, Czech Republic; 10 Section of Clinical Virology, Department of Medical Sciences, Uppsala University, Uppsala, Sweden; The University of Hong Kong, Hong Kong

## Abstract

Oseltamivir (Tamiflu®) is the most widely used drug against influenza infections and is extensively stockpiled worldwide as part of pandemic preparedness plans. However, resistance is a growing problem and in 2008–2009, seasonal human influenza A/H1N1 virus strains in most parts of the world carried the mutation H274Y in the neuraminidase gene which causes resistance to the drug. The active metabolite of oseltamivir, oseltamivir carboxylate (OC), is poorly degraded in sewage treatment plants and surface water and has been detected in aquatic environments where the natural influenza reservoir, dabbling ducks, can be exposed to the substance. To assess if resistance can develop under these circumstances, we infected mallards with influenza A/H1N1 virus and exposed the birds to 80 ng/L, 1 µg/L and 80 µg/L of OC through their sole water source. By sequencing the neuraminidase gene from fecal samples, we found that H274Y occurred at 1 µg/L of OC and rapidly dominated the viral population at 80 µg/L. IC_50_ for OC was increased from 2–4 nM in wild-type viruses to 400–700 nM in H274Y mutants as measured by a neuraminidase inhibition assay. This is consistent with the decrease in sensitivity to OC that has been noted among human clinical isolates carrying H274Y. Environmental OC levels have been measured to 58–293 ng/L during seasonal outbreaks and are expected to reach µg/L-levels during pandemics. Thus, resistance could be induced in influenza viruses circulating among wild ducks. As influenza viruses can cross species barriers, oseltamivir resistance could spread to human-adapted strains with pandemic potential disabling oseltamivir, a cornerstone in pandemic preparedness planning. We propose surveillance in wild birds as a measure to understand the resistance situation in nature and to monitor it over time. Strategies to lower environmental levels of OC include improved sewage treatment and, more importantly, a prudent use of antivirals.

## Introduction

The recent A/H1N1 pandemic has once again put the spotlight on influenza as a major human health problem. In a pandemic situation, preparedness plans rely heavily on vaccines and antiviral drugs. Vaccines are effective as prophylaxis, but the process of mass-scale production takes several months and therefore antiviral drugs are essential, especially during the first wave of a pandemic. A major and growing concern is the development of resistance to the few antiviral drugs available. Of these drugs, Oseltamivir (Tamiflu®) is most widely used. Resistance to oseltamivir was very rarely detected in clinical practice until the 2007–08 season when the mutation H274Y in the neuraminidase (NA) gene occurred. The H274Y mutation confers resistance to oseltamivir and was found in circulating seasonal H1N1 virus, initially in Europe [1] but the proportion of seasonal H1N1 carrying H274Y quickly increased and predominated worldwide in the 2008–09 season [2]. Oseltamivir usage did not correlate with the occurrence of resistant virus [2], [3], implying that the strain containing H274Y had a fitness comparable to other circulating viruses. After the A/H1N1 pandemic, this seasonal H1N1 virus has virtually disappeared from the strains circulating among humans. To avoid confusion, the virus is therefore denoted as “former seasonal H1N1”. The H274Y mutation has also emerged in patients with pandemic A/H1N1 influenza treated with oseltamivir [4], [5]. So far, the H274Y mutant is rare among pandemic A/H1N1 virus but could increase as some cases of suspected human-to-human transmission have been reported [6]. Fitness studies of resistant pandemic A/H1N1 have shown equivalent virulence in animal models [7], [8] but signs of decreased transmissibility in ferrets [7].

Oseltamivir is extensively stockpiled; e.g. the US had 40 million treatment courses in stock as of April 2009 [9]. Worldwide, more than 220 million courses have been stockpiled, and the shelf life has been extended to 7 years [10]. The active metabolite of oseltamivir, oseltamivir carboxylate (OC), is poorly absorbed from the gastrointestinal tract and the drug is therefore administered as a prodrug, oseltamivir phosphate (OP). OP is readily absorbed and rapidly converted to OC by esterases, and more than 75% of an oral dose reaches the circulation as OC. The active metabolite is then excreted in an unchanged form via the urine [11]. OC is stable in the aqueous phase and degrades poorly in sewage treatment plants (STPs) and surface water [12], [13]. Thus, there is reason to believe that OC is present in the aquatic environment near STPs when oseltamivir is used extensively. Japan has had the highest per-capita consumption of oseltamivir during several seasonal influenza outbreaks. For example, during the 2004/2005 season more than 10 million treatment courses were used, corresponding to almost 10% of the population [14]. Two studies have detected OC in surface water and outgoing water from STPs in Japan during seasonal influenza outbreaks [15], [16].

Dabbling ducks are the natural reservoir for influenza A viruses [17] and can be exposed to OC in the aquatic environment near STPs. An infection with influenza A virus in ducks results in mild clinical signs. Physiological data from an infection experiment showed only a transient, small increase in body temperature [18] and in another experiment mallard hens had a transient decrease in egg production [19]. Migrating dabbling ducks positive for influenza A had a 20 g lower mean weight than uninfected birds [20]. The infection of low-pathogenic avian influenza viruses in dabbling ducks is mainly gastrointestinal [21]. OC is poorly absorbed from the bowel in humans and it is likely that the absorption is poor also in ducks. However, the gastrointestinal location of the infection in dabbling ducks means that OC could directly affect replicating virus in the intestine.

**Figure 1:**
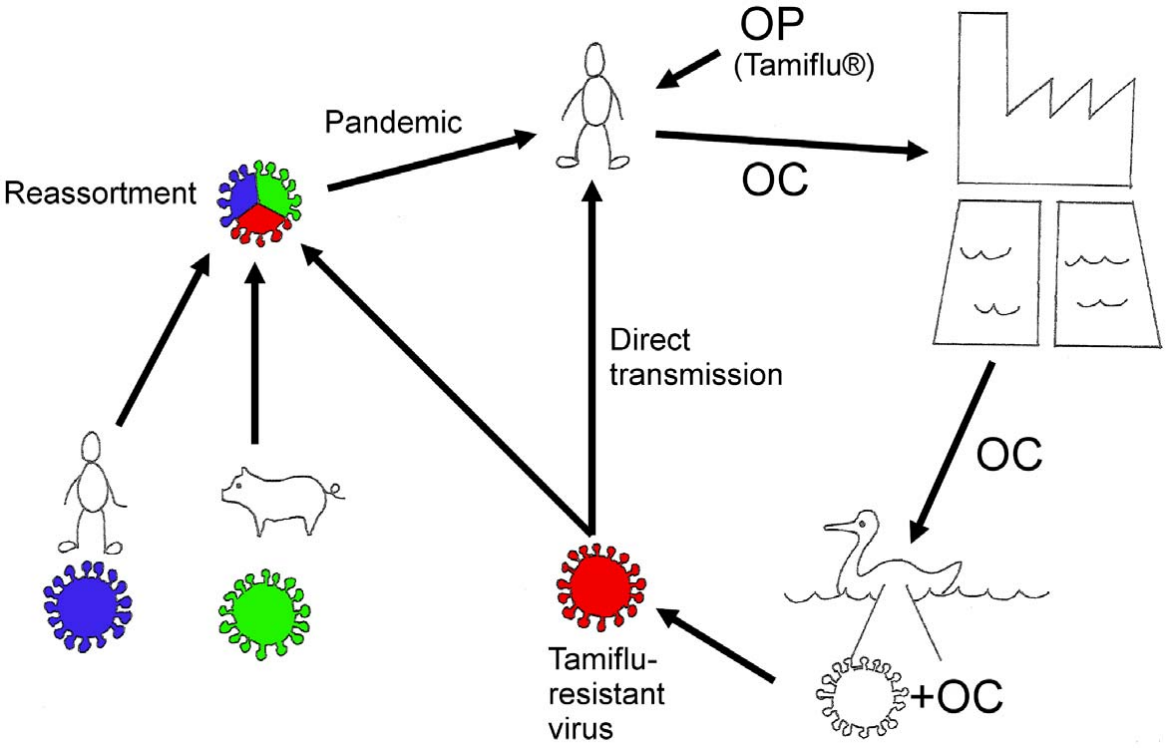
Summary of the general hypothesis. As OC is poorly degraded in STPs and surface water, it can enter aquatic environments where dabbling ducks can be exposed to the substance. Dabbling ducks are the natural influenza reservoir and have a perpetual circulation of influenza viruses in their population. Thus, there is a risk of resistance development in the bowel of the ducks where replicating virus and OC co-exist. Through reassortment or direct transmission, an oseltamivir-resistant influenza virus could spread to humans. Illustration by S.J. Järhult.

If resistance is established in influenza viruses circulating among wild birds, there is a risk of re-entry to humans, either via direct transmission or reassortment — this is further commented on in *Discussion*. Figure 1 displays a summary of the general hypothesis.

We hypothesized that influenza virus in mallards (*Anas platyrhynchos*) exposed to low levels of OC can develop resistance. Therefore, we infected mallard ducks with an avian influenza A/H1N1 virus isolated from a wild mallard and experimentally varied OC levels in the sole water source. Uninfected ducks were then successively introduced and the virus was transmitted serially from duck to duck to mimic the passing of influenza between ducks in the wild. Resistance development was assessed by sequencing the NA gene from daily fecal samples. Three experiments were performed at 80 ng/L, 1 µg/L and 80 µg/L of OC. Our results demonstrate that resistance develops through acquisition of the mutation H274Y.

## Results

### Mallard Model

Real-time reverse transcriptase PCR (q-PCR) on fecal samples confirmed that all ducks were negative for influenza A when transferred to the experiment room and that all ducks were infected with the A/H1N1 strain during the experiment. Viral shedding started day 1 post infection (pi) in most ducks and reached a maximum at day 2–3 pi. All ducks shed virus for 5 days. The 4 ducks at 80 µg/L of OC that were in the experiment for 7 days also shed virus intermittently day 6–7 pi. There was no difference in shedding pattern between ducks primarily infected with the viral stock solution and those where the infection was secondarily transmitted from another duck. Virus was detectable in the water samples from most days (69/73 evaluable samples) but in lower concentrations compared to the fecal samples.

### OC Analysis

Average concentrations of OC in the water from the sole water source of the ducks were measured at 83 ng/L ±23%, 0.95 µg/L ±24% and 81 µg/L ±11% (mean value and relative standard deviation) in the respective experiment. OC levels in the water were similar when measured immediately after addition of OC and before discarding the same water one day later (tested for three days during each experiment).

### OC Resistance

**Figure 2:**
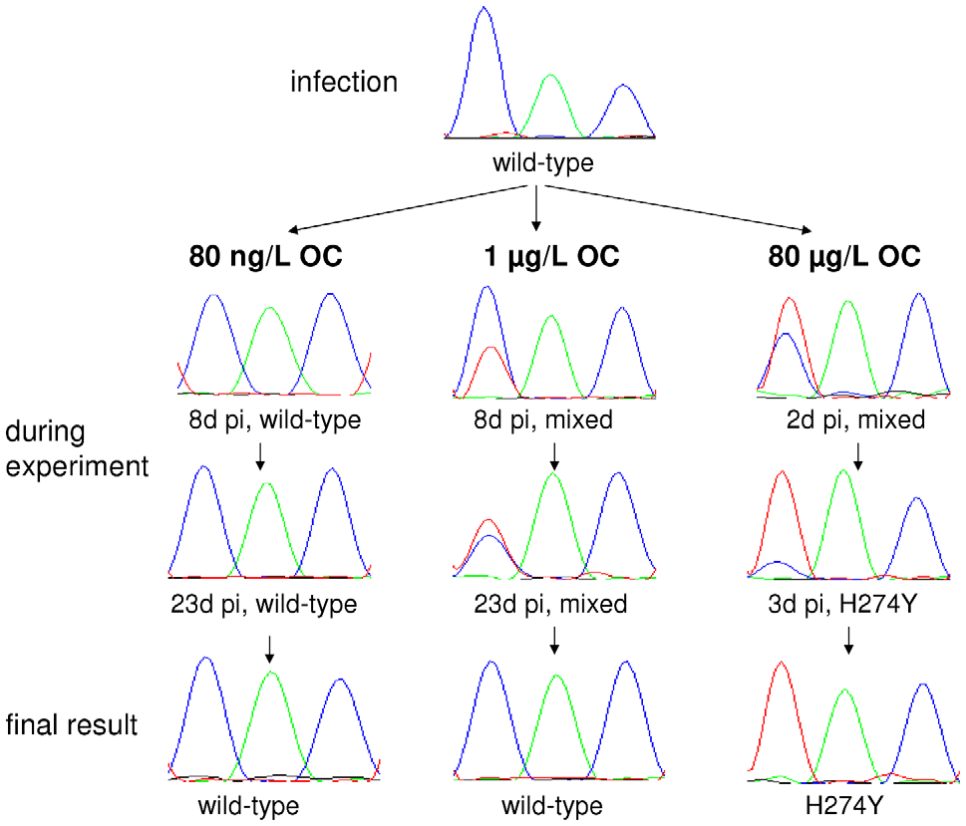
Genotypic results of experiments with 80 ng/L, 1 µg/L and 80 µg/L of OC. Sequencing results of the NA gene at amino-acid residue 274 (N2 numbering). Blue  =  cytosine (C), green  =  adenine (A) and red  =  thymine (T), corresponding to uracil (U) in the RNA sequence. Wild-type genotype  =  CAC  =  histidine, H274Y  =  TAC  =  tyrosine.

In the 80 ng/L experiment H274Y was not detected in any of the 134 evaluable samples. However, in the 1 µg/L experiment, 2 of 127 samples contained a mixture of genotypes consisting of both wild-type and H274Y strains (Figure 2). Those samples were from days 8 and 23 after the start of the experiment (second and seventh duck generation). The occurrence of both genotypes was confirmed by re-sequencing the samples twice, including a new RNA extraction from the original fecal sample. In the 80 µg/L experiment, sequencing of fecal samples showed H274Y 2 days pi and from 3 days pi and onwards, only H274Y was present in all 21 samples.

**Figure 3:**
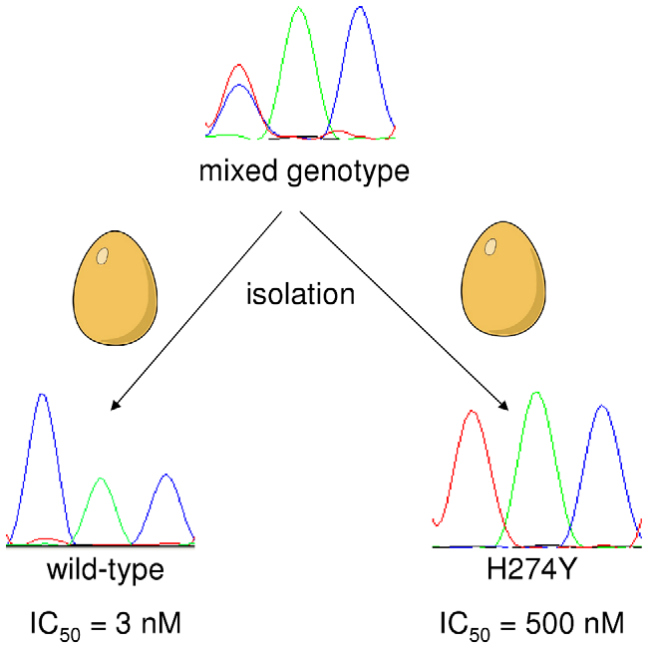
Different results of two isolations of a sample with mixed genotype. The mixed-genotype sample from day 23 pi in the 1 µg/L OC experiment was isolated twice in embryonated hen eggs. One of the isolations yielded a wild-type virus and one an H274Y mutant with corresponding IC_50_-values.

The 50% inhibitory concentration (IC_50_) of 13 tested wild-type viruses from the experiment was 2–4 nM whereas the IC_50_ of 15 tested H274Y isolates was 400–700 nM. Two different isolations of the same sample with mixed genotypes resulted in either the wild-type or the H274Y sequence with a corresponding IC_50_ (see Figure 3). The A/*Mallard*/Sweden/51833/2006(H1N1) strain used for inoculation had an IC_50_ of 3 nM.

### Analysis of Sequences from NCBI

See Table 1.

**Table 1:** Wild-type and H274Y-containing avian influenza A viruses of N1 subtype in the NCBI database.

	H274Y	Wild-type	Total
H1N1	1	208	209
H5N1	4 (3)*	2132	2136 (2135)
(other H)N1	0	459	459
Total	5 (4)	2799	2804 (2803)

* indicates that the four H274Y-positive H5N1 viruses probably represent three distinctly different viruses, see *Discussion*.

## Discussion

In an experimental set-up, we demonstrated that oseltamivir resistance develops through the acquisition of H274Y when mallards infected with influenza A/H1N1 virus were exposed to 1 µg/L of OC, and that the H274Y mutation rapidly dominated the viral population at an OC concentration of 80 µg/L. Previous reports of environmental OC levels during seasonal influenza outbreaks in Japan range from 58–293 ng/L [15], [16]. In a pandemic situation OC levels are expected to rise considerably, reaching the same magnitude as the levels where resistance development was observed in this study.

Oseltamivir resistance was confirmed functionally by an enzyme activity (MUNANA) assay. The more than hundred-fold higher IC_50_ among H274Y positive isolates is consistent with data from human clinical isolates [22]. Isolation of a sample with mixed genotypes can result either in a wild-type or in a H274Y-positive virus, which demonstrates that either genotype can dominate the replication during the isolation process. As there is no drug pressure when virus replicates in hen eggs, this indicates that the viral fitness is not dramatically different in the H274Y mutant compared to the wild-type virus.

The global spread of former seasonal H1N1 viruses carrying H274Y has shown that virus with this mutation can outcompete wild-type virus without drug pressure [2]. It has recently been demonstrated that this is probably due to “permissive” compensatory mutations e.g. R222Q and V234M, that restore the decreased surface expression of NA caused by H274Y [23]. By analogy, H274Y acquired in wild ducks during a temporary increase in environmental levels of OC could prevail even when oseltamivir is no longer widely used. As dabbling ducks are the natural reservoir for influenza A viruses [17], the circulating viral gene pool is large and variable. An example of the variable avian gene pool is a study where the sensitivity to OC in avian A/H1N1 viruses showed a much larger variation as compared to mammalian viruses [24]. As one of the few circulating former seasonal human A/H1N1 strains could adapt to H274Y it seems likely that some of the many circulating avian strains have the right genetic makeup to harbor H274Y without losing viral fitness. Compared to a randomly selected former seasonal A/H1N1 strain containing H274Y (A/*Norway*/1736/2007), the A/H1N1 strain used in this study had a protein homology of 84%. When compared to a wild-type pandemic A/H1N1 strain (A/*Georgia*/NHRC0001/2011), the protein homology was higher (90%) indicating a closer similarity. This is consistent with the fact that the pandemic A/H1N1 virus was derived from swine viruses that have a recent avian origin [25] while the former seasonal A/H1N1 virus has been circulating among humans for many years. The studied A/H1N1 strain did not contain R222Q or V234M. Thus, there is a lower probability that H274Y would be sustained without drug pressure if the virus does not contain other mutations increasing the surface expression of NA [23]. However, the A/H1N1 strain used in this study contained the mutation R222N. Although R222N is not previously described as a permissive mutation, Q and N have very similar uncharged polar side chains, suggesting that this mutation could have permissive capacities. H274Y emergence under drug pressure has been observed both in early treatment studies of former seasonal A/H1N1 with oseltamivir [26] and in pandemic A/H1N1 [4], [5]. Therefore, it is not surprising that H274Y emerges in the studied A/H1N1 strain although established permissive mutations are missing.

Little is known about the resistance situation for influenza A viruses in nature. The few H274Y-positive sequences found when screening the NCBI database (Table 1) indicate that the mutation is not yet common in nature. The two NA sequences from the H5N1 viruses isolated from swans in Aztrakhan, Russia, 2005 are identical in sequence, either being two submissions of the same virus or two samples from different birds carrying the same virus. Thus, the four H5N1 viruses positive for H274 probably represent three different viruses. The other two H274Y-positive H5N1 viruses are from a mute swan (*Cygnus olor*) in the Caspian Sea from 2006 and a chicken in Hong Kong from 2002. Interestingly, an H274Y-positive H1N1 virus was recently published in NCBI (22^nd^ December 2010) and it has not yet been commented on in a journal article. This virus was obtained from a 2007 sample of a duck in Minto Flats in Interior Alaska, a habitat with high densities of nesting ducks. Thus, H274Y-positive avian N1 viruses can be fit enough to occur in the wild, both in highly pathogenic H5N1 and in low-pathogenic H1N1 viruses without obvious drug pressure. Furthermore, it has recently been demonstrated by our group that resistance mutations to neuraminidase inhibitors occur also among influenza viruses isolated from wild birds in Sweden [27]. These viruses were obtained from dabbling ducks in an environment where no OC is present. Taken together, these observations demonstrate that H274Y can exist among influenza viruses in wild birds also when OC is absent. Thus, there is a possibility of resistance accumulation when oseltamivir is widely used, even if OC disappears from the environment in between influenza outbreaks. We therefore propose surveillance in wild bird populations as an important measure to gain more knowledge of the resistance situation in nature and to monitor it over time.

The *in vivo* mallard model described here provides a promising means to study viral evolution under dynamic conditions, e.g. drug pressure. It is possible to control the experimental conditions, yet the virus is subjected to both replication and transmission as in the natural situation. The similar shedding patterns in ducks primarily infected by inoculation and secondarily infected by transmission and the finding that all ducks introduced were infected demonstrated that the natural route of transmission used in the model is effective also in an experimental setting. The timing of the introduction of new birds at day 3 pi seems logical as ducks shed the highest levels of virus day 2 and 3 pi. Considering the low levels used, the water concentrations of OC were constant and there were no signs of OC degradation during the one-day use of each OC/water mixture. This is consistent with the fact that OC is poorly degraded in surface water [12], [13]. Possible future uses of the mallard model, apart from drug exposure studies, include persistence analysis of resistant viruses and *in vivo* testing of viruses isolated from wild birds.

Strategies to reduce environmental levels of OC include improved sewage treatment—some bioremeditative effect has been shown by a granular bioplastic formulation of the fungus *Phanerochaete shrysosporium* [28]. Furthermore, two bacterial strains growing on OC as the sole carbon source has been isolated from the sediment of Japanese rivers [12] and ozonization to lower OC levels has been discussed [15]. However, to substantially reduce the amount of OC in the environment, a prudent use of antivirals is vital. In critically ill or immunosuppressed patients there is growing evidence that a combination therapy approach can be favorable in preventing resistance development in the treated patient [29], [30]. However, these patients constitute a small minority and by restricting other uses in non-pandemic periods, we can save the antivirals until needed.

Influenza A viruses can cross species barriers either by direct transmission or by genetic reassortment with other influenza viruses. An alarming scenario of direct transmission is if a high-pathogenic avian influenza virus (e.g. H5N1) acquires the H274Y mutation and then adapts to humans. By reassortment, an avian influenza virus with an NA gene containing H274Y could combine with human and other mammalian influenza viruses resulting in an oseltamivir-resistant pandemic strain. Viruses from all four influenza pandemics during the last century contain avian genetic material [25]. Regardless of whether the route is by direct transmission or by reassortment, the H274Y or other resistance mutations can spread from birds to humans, thus disabling oseltamivir, a cornerstone in pandemic preparedness planning.

In conclusion, our work demonstrates a hitherto unknown setting for resistance development in influenza A. Environmental levels of the active metabolite of the antiviral oseltamivir can induce acquisition of the resistance mutation H274Y in influenza A/H1N1 virus in mallards. There is a risk that H274Y, or other resistance mutations, will be spread among the plethora of influenza A viruses circulating in nature, including those with pandemic potential. As oseltamivir is stockpiled worldwide as a cornerstone in pandemic preparedness plans, the threat of an oseltamivir-resistant influenza pandemic has major implications in a general public health perspective.

## Materials and Methods

### Animals and Housing

This study was carried out in strict accordance with recommendations from the Swedish Board of Agriculture. The protocol was approved by the Ethical Committee on Animal Experiments in Uppsala (permit C82/09). All efforts were made to minimize suffering for the animals. Male mallard ducks were purchased from a Swedish farm at the age of 3–6 months and then kept at the National Veterinary Institute (Uppsala, Sweden). All mallards initially tested negative for influenza A using q-PCR (described below, [31]) and serology using a nucleoprotein-targeting ELISA assay (Avian Influenza A Blocking ELISA, Pourquier, France).

### Virus Preparation

The A/*Mallard*/Sweden/51833/2006(H1N1) strain (GenBank accession number JF710317) was isolated from a wild mallard sampled at Ottenby, Sweden. Virus isolation was performed by inoculating 200 µl of the sample medium in the allantoic cavity of 10-day-old embryonated hen eggs. The allantoic fluid was harvested three days later, centrifuged and virus growth was confirmed by a standard hemagglutination test. Virus stock for duck inoculation was obtained by a second passage, where diluted allantoic fluid from the primary isolation was inoculated and grown in the same fashion. The viral titer was determined by 50% Embryo Infectious Dose (EID_50_) [32]. The NA gene of the virus stock was sequenced in the same way as experimental samples (see below) and no known markers of resistance were observed. The NA sequence was used as a reference to monitor NA changes during the experiment.

### Experimental Set-up

**Figure 4:**
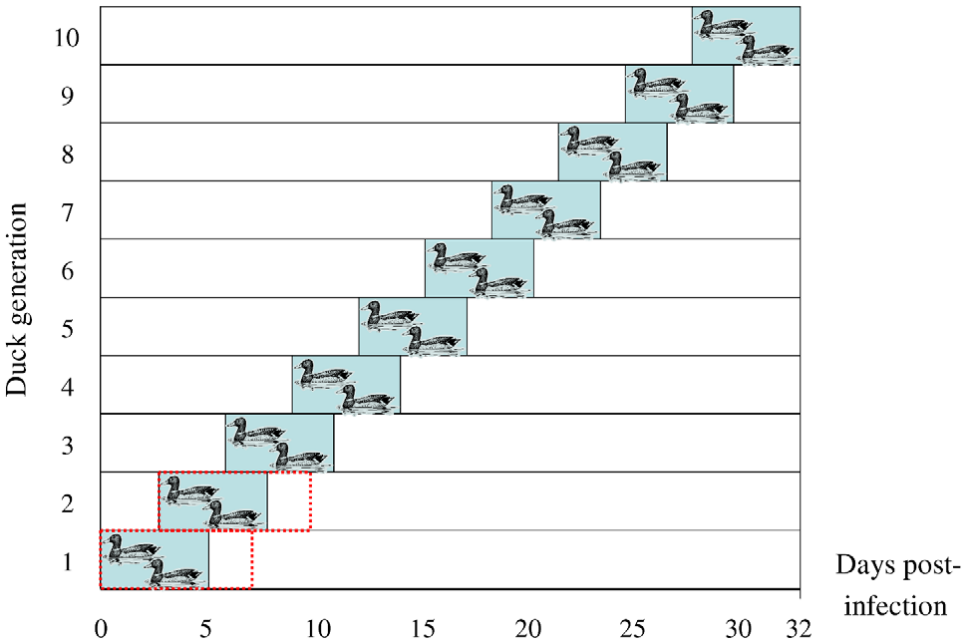
Experimental set-up. Set-up for 80 ng/L and 1 µg/L experiments shown with blue blocks, 80 µg/L with red dotted lines.

Primary infection was performed by esophageal inoculation of 1 ml of viral stock solution corresponding to 10^8^ EID_50_. Prior to the experiment, the ducks were kept in a separate room. A 1 m^2^ pool was the sole water source in the experiment room. The water was changed daily and OC was added; the final concentrations in the different experiments were measured at 80 ng/L, 1 µg/l and 80 µg/L, respectively. The 80 ng/L and 1 µg/L experiments consisted of ten duck generations, each lasting five days (see Figure 4). New ducks were introduced every third day and kept together for two days before the preceding generation was removed. In the 80 µg/ml experiment, two seven-day generations were used. Each generation consisted of two ducks. To minimize the risk of the next generation being infected by remaining virus instead of the intended direct transmission, the room was thoroughly cleaned before the introduction of a new generation.

### Sampling and Virus Detection

When daily sampling was performed, ducks were put into separate clean cardboard boxes. Fecal samples were obtained either by cloacal swabbing or by swabbing of fresh feces from the boxes. The samples were put in 1 ml of transport medium as described earlier [33] and stored at −80°C. The water samples for virus detection and OC analyses were taken daily and were stored at −80°C and −20°C respectively.

### Q-PCR

A previously published one-step real-time reverse transcriptase PCR [31] targeting the matrix gene was used to monitor the infection on a daily basis. The reaction was performed in a Corbett Research Rotor-Gene 2000 Real-time ThermoCycler (Corbett Research, Mortlake, Australia), using iScript one-step RT-PCR kit for probes (Bio-Rad, Hercules, USA). A total reaction volume of 25 µl was used, containing 12.5 µl 2x RT-PCR reaction mix for probes, 2.5 µl of RNA extract and final concentrations of 125 nM of the probe, 0.5 µl of iScript reverse transcriptase and 200 nM of each forward and reverse primer.

### Sequencing

134 sequences of H1N1 NA retrieved from GenBank were used for multiple sequence alignment using BioEdit V7.0.9.0 [34]. Three pairs of primers were designed to cover the full-length NA gene as shown in table 2. All primers were purchased from Thermo Hybaid, Interactiva Division (Ulm, Germany).

**Table 2:** Primers designed for PCR amplification and sequencing of the neuraminidase (NA) gene.

Primer no.	Sequence (5′- 3′)
1	GCAGGAGTTCAAAATGAATCCAAATC
2	TGTTCAAAAAACTCCTTGTTTCTACT
3	CCATTGGGTCAATCTGTATGGTGA
4	GTTGCCATTCACCATTGACAAGTAGT
5	CTCATGCTCCCACTTGGAAT
6	GTGTCCTCTAACGGGGCATA

RNA was isolated using the Magnatrix 8000 extraction robot (Magnetic Biosolutions, Sweden) and Vet Viral NA kit (NorDiag ASA, Oslo, Norway). PCR amplification of the NA gene was performed using SuperScript III One-Step RT-PCR System with Platinum Taq High Fidelity (Invitrogen) with primers 1 and 2. The total reaction volume of 25 µl contained 12,5 µl of 2x reaction buffer, 0,5 µl of Platinum Taq High Fidelity enzyme, 5 µl of RNA extract and forward and reverse primers to a final concentration of 200 nM each. Some samples required an additional NA PCR with a doubled reaction volume. The NA PCR-products were either gel purified (Qiaquick PCR purification kit; Qiagen) or purified using ExoSAP-IT treatment (Affymetrix). The purified PCR products were sent to Macrogen, Korea, for sequencing. Sequences were analyzed using SeqScape software (Applied Biosystems).

### NA Inhibition Assay (NAIA)

Selected samples were grown in embryonated hen eggs, as described in Virus Preparation. NA enzymatic activity was then measured using the fluorogenic substrate 2′-(4-methylumbelliferyl)-α-D-N-acetylneuraminic acid (MUNANA; Sigma) [35]. The fluorescence of the released 4-methylumbelliferone was measured using a GloMax multiplate reader (Promega) at excitation and emission wavelengths of 350 and 450 nm, respectively. For NAIA, viral suspensions were adjusted to equivalent NA contents in MES buffer (32.5 mM morpholineethanesulfonic acid, pH 6.5, 120 mM NaCl, 4 mM CaCl2), based on preliminary determinations of the NA activities in serial dilutions of the viral stocks. OC (RO0640802-002; lot: 01007B243804) was obtained from Roche (F. Hoffmann-La Roche Ltd, Basel, Switzerland). Viral suspensions were preincubated in the presence of various concentrations of OC (0.015 to 4000 nM) for one hour at 37°C in 96-well plates, with shaking. Following the addition of substrate at a final concentration of 100 µM, the viruses were incubated for one hour at 37°C, and the reaction was stopped by adding one volume of a solution of 1 M glycine, pH 10.7, and 25% ethanol. Fluorescence values were measured, and the IC_50_ for NA enzymatic activity was determined from the dose-response curve, using GraphPad Prism Version 5 software (GraphPad software). IC_50_ values stated are means of duplicate experiments.

### OC Analysis

Two different arrangements of liquid chromatography-tandem mass-spectrometry (LC-MS/MS) were used to analyze pre-filtered (0.45 µm filters) water samples (0.5–10 ml; sample volumes were adjusted to the exposure levels in the experiment) collected during the three experiments. Water samples collected during the 80 ng/L experiment were analyzed using an on-line LC/LC-tandem mass-spectrometry (MS/MS) system including a PAL HTC autosampler (CTC Analytics AG, Zwingen, Switzerland), a Surveyor LC-Pump (Thermo Fisher Scientific, San Jose, CA, USA) connected to a extraction Hypersil GOLD column (20 mm×2.1 mm i.d. ×12 µm particles, Thermo Fisher Scientific, Waltham, MA, USA) and an Accela LC pump (Thermo Fisher Scientific, San Jose, CA, USA) connected to an analytical Hypersil GOLD aQ column (50 mm ×2.1 mm i.d. ×3 µm particles, Thermo Fisher Scientific, San Jose, CA, USA) with a guard column (2 mm ×2 mm i.d. ×3 µm particles, same packing material and manufacturer as the analytical column) coupled to a triple stage quadrupole MS/MS TSQ Quantum Ultra EMR (Thermo Fisher Scientific, San Jose, CA, USA). The water samples collected during the 1 µg/L and 80 µg/L experiments were analyzed by direct injection onto the analytical Hypersil GOLD aQ column using the conventional LC-MS/MS part of the system described above. In both analyses, heated electrospray (HESI) in positive ion mode was used for ionization of OC and two SRM transitions were monitored (285→138, collision energy 20 V; 285→120, collision energy 30 V). The level of OC in the samples was quantified using internal standard method (isotopic labeled OC obtained from Roche, F. Hoffmann-La Roche Ltd, Basel, Switzerland, was used as internal standard) with five calibration points. The maximum difference between results at quantification (285→138) and qualification (285→120) mass transition was set to 20% as criterion for positive identification.

### Analysis of NA Sequences from NCBI

On Jan 27, 2011, the NCBI Influenza Virus Sequence Database [36] was screened for all avian influenza viruses of the N1 subtype. Sequences were aligned using Muscle software [37] and viewed in BioEDIT version 7.0.9.0.
